# Treatment of the surgical neck fracture of the humerus with a novel external fixator in the elderly with osteoporosis: biomechanical analysis

**DOI:** 10.1186/s12891-019-2599-8

**Published:** 2019-05-15

**Authors:** Haijing Huang

**Affiliations:** Department of trauma Orthopedic, Tianjin Hospital, No. 406, Jiefang south Road, Hexi District, Tianjin, 300021 China

**Keywords:** Surgical neck fracture of humerus, External fixator, Plate, Load bearing, Resistance to torsional stress

## Abstract

**Background:**

No consensus exists on the treatment of proximal humerus fractures, especially in the elderly patients with osteoporosis. This study was aimed to evaluate the biomechanical characteristics of a novel external fixator in treating two-part surgical neck fracture of the proximal humerus in the elderly patients with osteoporosis.

**Methods:**

Sixteen female elderly humeral shaft specimens with osteoporosis were randomized into 2 groups. Models with the surgical neck two-part fracture of the proximal humerus were built, in which a novel external fixator (test group) and a clover plate (control group) were applied separately. In the test group, the fracture was firstly fixed with intersection pinning using 3 Schanz pins (3.5 mm), followed by the novel external fixation frame. In the control group, a clover plate and 6 cortical bone screws were applied. Biomechanical testing of the specimens was performed to assess the resistance to load bearing and torsional stress. The parameters of the two groups were compared using independent t-test.

**Results:**

Ultimate bearing capacity and load bearing at different parts with the humerus rotation were higher (*P* < 0.05) in the external fixator group (145.16 ± 17.42 N and 140 N respectively) than those in the plate group (120.21 ± 13.15 N and 69.63 ± 25.16–90.78 ± 17.18 N respectively). As for resistance to torsional stress, plate’s torque fluctuated between 1 Nm and 5 Nm, while the external fixator’s torque values were more evenly (*P* < 0.01) distributed with the fluctuation within 1 Nm.

**Conclusions:**

In the fixation of two-part humeral fracture in elderly patients with osteoporosis, the new external fixator seemed to be superior to plate fixation in load bearing and resistance to torsional stress.

## Background

Proximal humerus fractures are very common and often accompanied by osteoporosis. It has an increasing incidence with the increasing aging population in China [1, 2]. Recent epidemiological survey revealed that the annual incidence of proximal humerus fracture was as high as 2.48/1000 [3]. According to the Neer classification, 28% patients have two-part fracture and it mostly occurs in the patients over 50 years old, with a mean age of 72 years old [4, 5]. Although some studies have compared the treatments results between intramedullary pinning, plate fixation and percutaneous fixation, no consensus has been reached [6]. Every method has advantages and drawbacks. Plate fixation tends to achieve better biomechanical stability. However, it requires extensive surgical exposure, thus it is prone to complications of soft tissues as well as aseptic necrosis of the humeral head [7]. Antegrade intramedullary pinning can provide fracture reduction fixation with minimal invasion, but it may induce rotator cuff injury [8]. Percutaneous pinning is characterized by minimal invasion and low cost, but its biomechanical stability is not satisfactory, which may result in loss of reduction position and withdrawal of internal fixation.

The percutaneous pinning technique represents a minimally invasive procedure, which avoids extensive exposure and complications. Screw pin fixation has more remarkable advantages, although about 27% patients may still display loss of fracture contraposition, which is mainly caused by fixation failure due to old age and osteoporosis [9]. It is reported that percutaneous pinning can be applicable in elderly patients with satisfactory efficacy [10]. Rogner et al. proposed that minimally invasive fixation with percutaneous pinning should be ideal for treating elderly patients whose main demand is functional recovery of the joint under painless circumstances rather than restoring muscle strength [11].

Therefore, this study was designed to evaluate the biomechanical characters of external fixator and closed intersection pinning in treating two-part surgical neck fracture of humerus in elderly patients with osteoporosis. The biomechanical characteristics including load bearing and torsional resistance were assessed. The treatment combining internal fixation and plates was used as control.

## Methods

### Specimen

Sixteen female humerus specimens (average age of 73 ± 3, range of 69–83 years old) provided by Tianjin Hospital were used in this study. Those specimens had no congenital malformations, fractures, and tumors. All specimens were subjected to deep cryopreservation (− 100 °C).

The specimens were thawed at room temperature at 12 h before tests. Bone mineral density of humeral shaft was measured. The same regions of those humeral heads were delineated and the bone mineral density was assessed by Dual-energy X-ray absorptiometry (QDR-4500A ACCLAIM, HOLOGIC, United States). The samples were confirmed to meet the WHO standards of osteoporosis (BMD between 0.2 and 0.4 g/cm^2^) [12].

### Construction of fracture model

Those specimens were randomized into control group (clover plate) and test group (external fixator) using random table method. Models of surgical neck two-part fracture of the humerus were constructed according to the previous reports [13, 14]. Osteotomy of the humerus surgical neck was performed in humeral shaft specimens using an electric oscillating saw with a 1 mm-thickness saw bit. A horizontal reference line was drawn at 3 cm below the apex of the greater tubercle and it crossed the base of the lesser tubercle and constituted 20° with the osteotomy line. All the fracture models were built by the same person.

### Fixation procedures

**Fig. 1:**
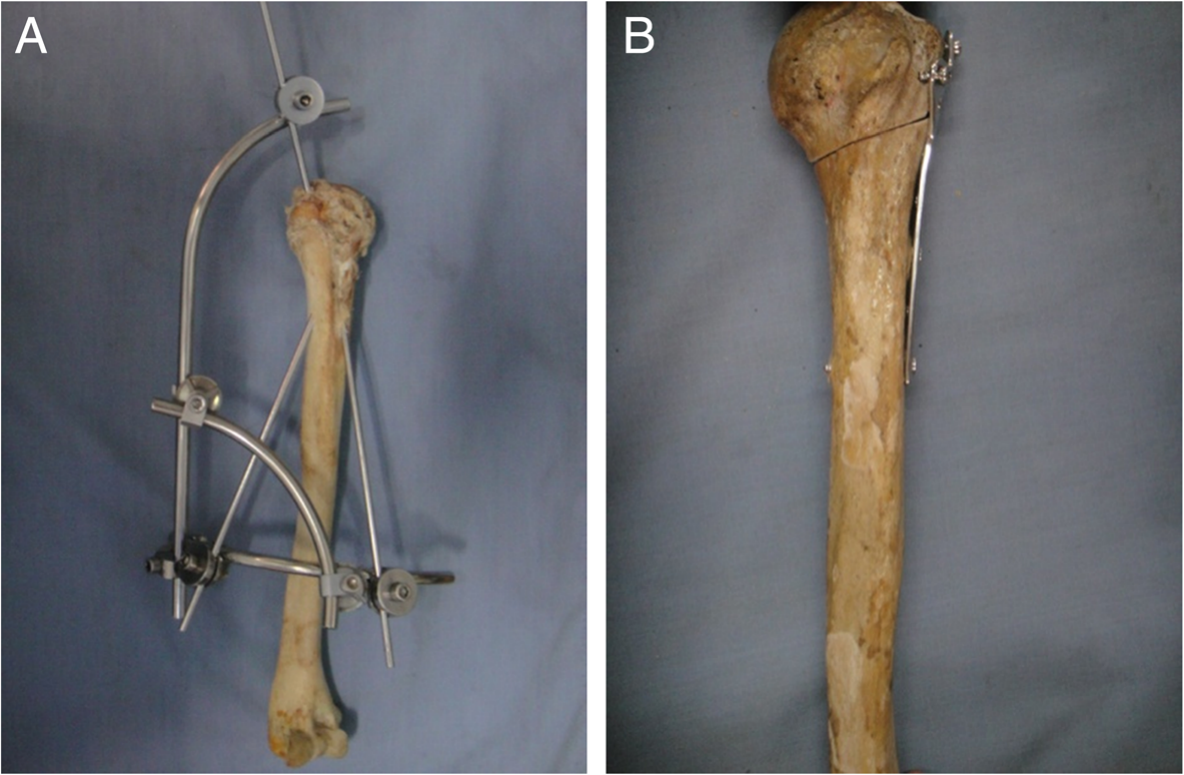
Intersection pinning and plate fixation (**b**) for the surgical neck fracture of the humerus. **a**, in the external fixator group, three driving screws were locked with fixed links and clamps. **b**, in the plate fixation group, the end of the humeral shaft was fixed with 3 cortical screws (3.5 mm) and that of humeral head fixed with 3 cancellous screws (4.0 mm)

**Fig. 2:**
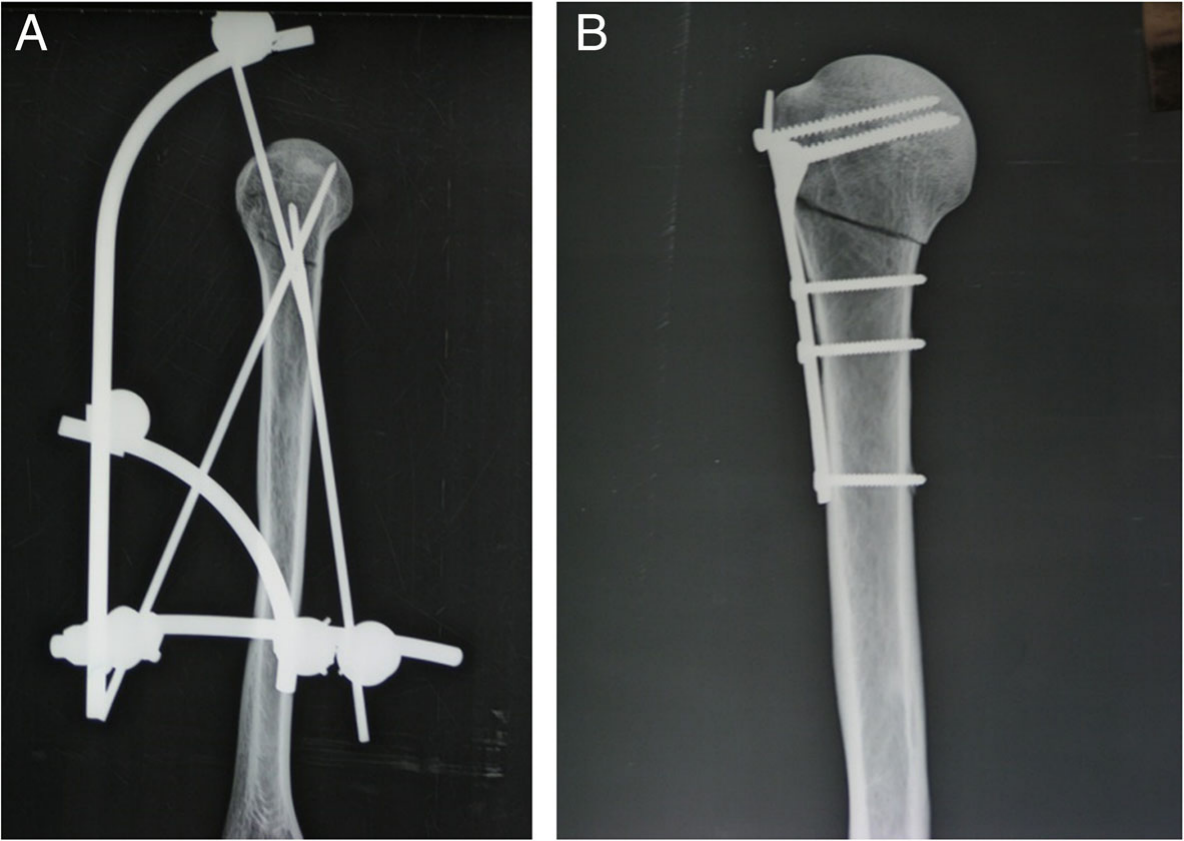
Lateral X-ray images of the external fixator (**a**) and plate fixation (**b**)

The procedures in external fixator group were done under fluoroscopy as the following. The parts situated 2 cm above the lateral humeral epicondyle were vertically removed. Denture acrylic was embed vertically The surgical neck fracture was made in the proximal humerus with a handsaw. The intersection between the deltoid muscle’s insertion plane and lateral edge of the caput longum musculi bicipitis brachii was used as the entrance point of the first fixation pin (Schanz nail, 3.5 mm × 150 mm; Tianjin Xinzhong Medical Devices Co., Ltd. China). The insertion direction was backward tilted for 20–30° from anterolateral to posteromedial normal humeral head, whereby the humeral head center was located at posterior humeral shaft. For anterolateral insertion, the pin formed 45° in the coronal plane with the humeral shaft, and formed 30° in the sagittal plane with the humeral shaft. Thus, the insertion point was located below the humeral head center at 0.5-1 cm below the humeral head. The second pin was placed in front of the humeral shaft, and was inserted anteroposteriorly in the insertion plane of the deltoid muscle, where it formed 45° with the humeral shaft in the sagittal plane and formed 30° in the coronal plane. Therefore, the insertion point was located at the posterosuperior part of the humeral head. The third pin was located at the apex of the greater tuberosity of humerus, forming 30° with the sagittal plane of the humeral shaft. Considering that a driving screw reached the medial cortex of the humeral shaft beyond the fracture line, three driving screws (Tianjin Xinzhong Medical Devices Co. Ltd., Tianjin, China) were locked with fixed links and clamps (Figs. 1 and 2).

The surgery in the clover plate group was done through anterior approach. Trilobal plates (non-locking; Kanghui Ltd. Co., Changzhou, China) were placed at 1 cm below the lateral apex of the greater tubercle of the humerus. Clover plates (Kanghui Ltd. CO., Changzhou, China) were internally fixed. The end of the humeral shaft was fixed with 3 cortical screws (3.5 mm) and that of humeral head was fixed with 3 cancellous screws (4.0 mm) (Figs. 1 and 2). All above fixation procedures were done by the same person.

### Measurement

**Fig. 3:**
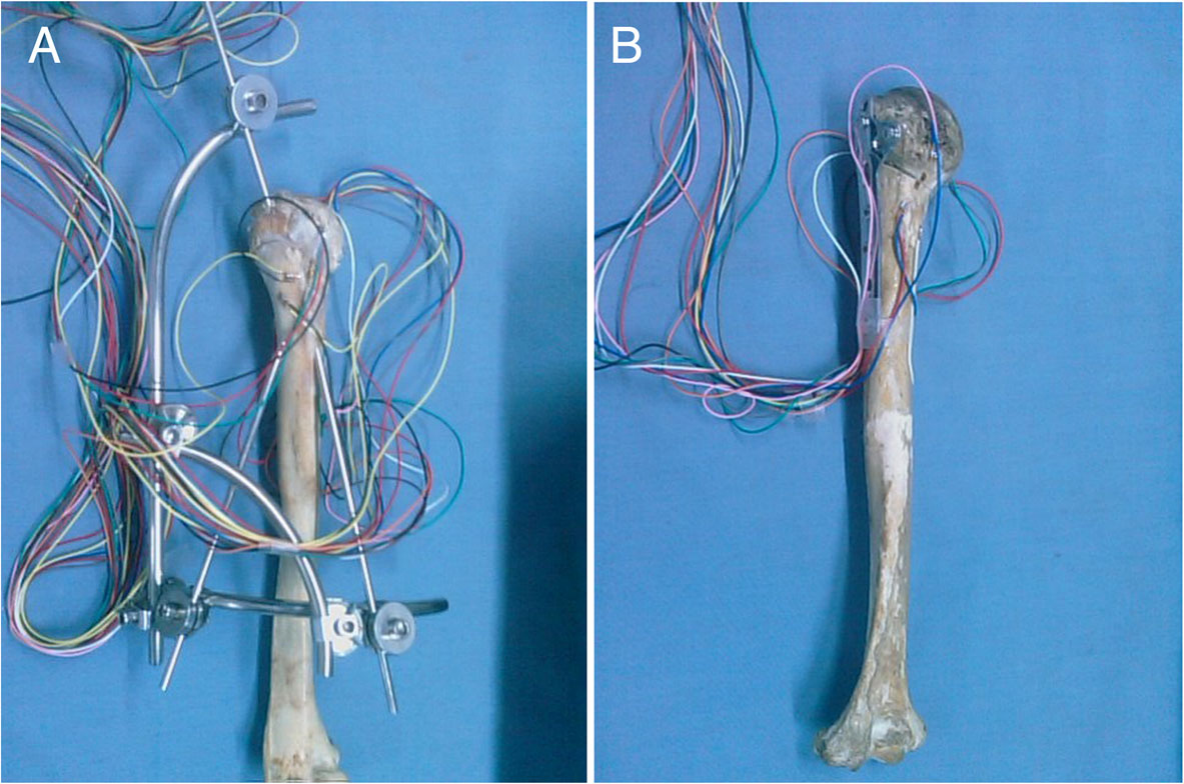
Specimens with external fixation (**a**) and plate fixation (**b**) for measurement of the resistance to load and torsional stress. Ten strain gage pieces were attached along the two sides of the fracture line, with a vertical distance of 2 mm to the broken site

The measurement was performed by reference to the previous reports. [15, 16] Ten strain gage pieces were attached along the two sides of the fracture line, with a vertical distance of 2 mm to the broken site. BE120-05AA-X30 foil gages (Hanzhong Zhonghang Electronic Measuring Instruments, China) were used at 120 ± 0.1 Ω resistance and a sensitivity coefficient of 1.94% ± 1.00%. Then, resistance fluctuation was assessed using YJ-33 static resistance strain indicator (Shanghai Automation Instrumentation Co., Ltd., Shanghai, China). Strain gage resistance might present a small variation (< 1 Ω) after the attachment (Fig. 3).

**Fig. 4:**
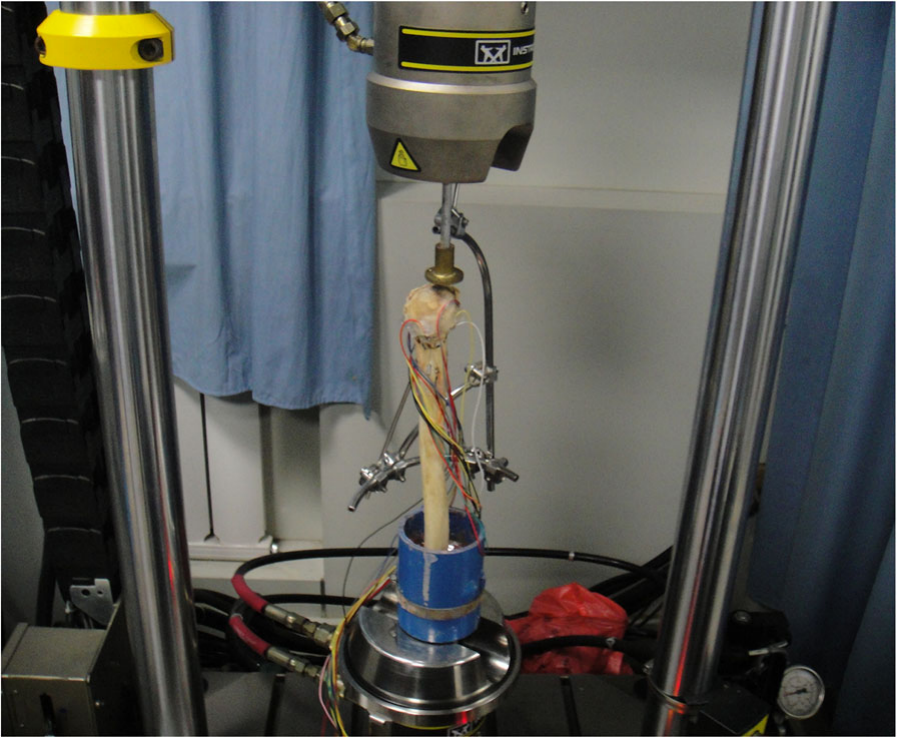
Compression and torsion experiments and data acquisition for the external fixator group. A gradually-increasing load from 0 to 200 N was applied at a loading rate of 1.4 mm/min. The values of multiple strain gages at both sides of the broken ends were simultaneously acquired. The specimens were rotated for 6 cycles or until damaged

**Fig. 5:**
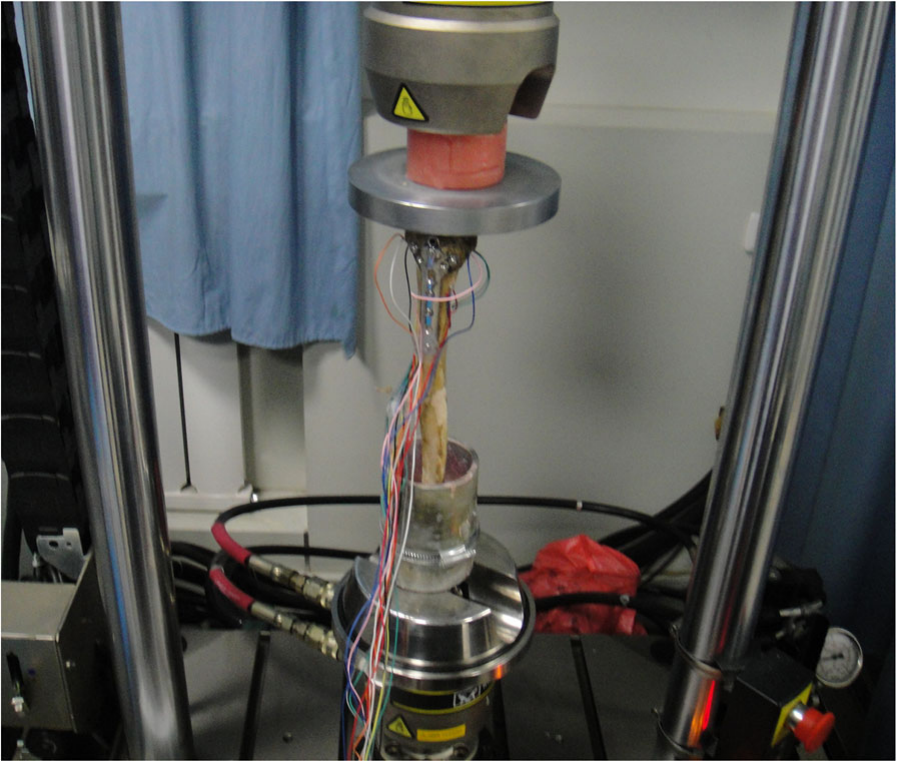
Compression and torsion experiments and data acquisition for the clover plate group. A gradually-increasing load from 0 to 200 N was applied at a loading rate of 1.4 mm/min. The values of multiple strain gages at both sides of the broken ends were simultaneously acquired. The specimens were rotated for 6 cycles or until damaged

The fracture models were submitted to compression resistance test at 25 ± 1 °C and 30% humidity. To simulate the loading status of human shoulders, the specimens were vertically placed on the hydraulic servo dynamic biomechanical tester (Instron 8874, USA), with the distal ends completely fixed on the lower part of the tester. The upper clutch disk was adjusted to fully contact the distal end of specimens. A gradually-increasing load from 0 to 200 N was applied at a loading rate of 1.4 mm/min. The values of multiple strain gages at both sides of the broken ends were simultaneously acquired. Compression and torsion experiments were performed to determine the loads and torques by reference to the previous reports. [15, 16] Data acquisition time was 50s, and the specimens were rotated for 6 cycles or until damaged. Data acquisition for the fixations is shown in Figs. 4 and 5.

### Statistical analysis

SPSS 21.0 software (SPSS, Chicago, IL, USA) was used. The average values, expressed as mean ± standard deviation, were used for statistical analysis. The comparison of the measures between the control group and the test group was performed using independent t-test. *P*-value < 0.05 was considered as statistical significance.

## Results

**Fig. 6:**
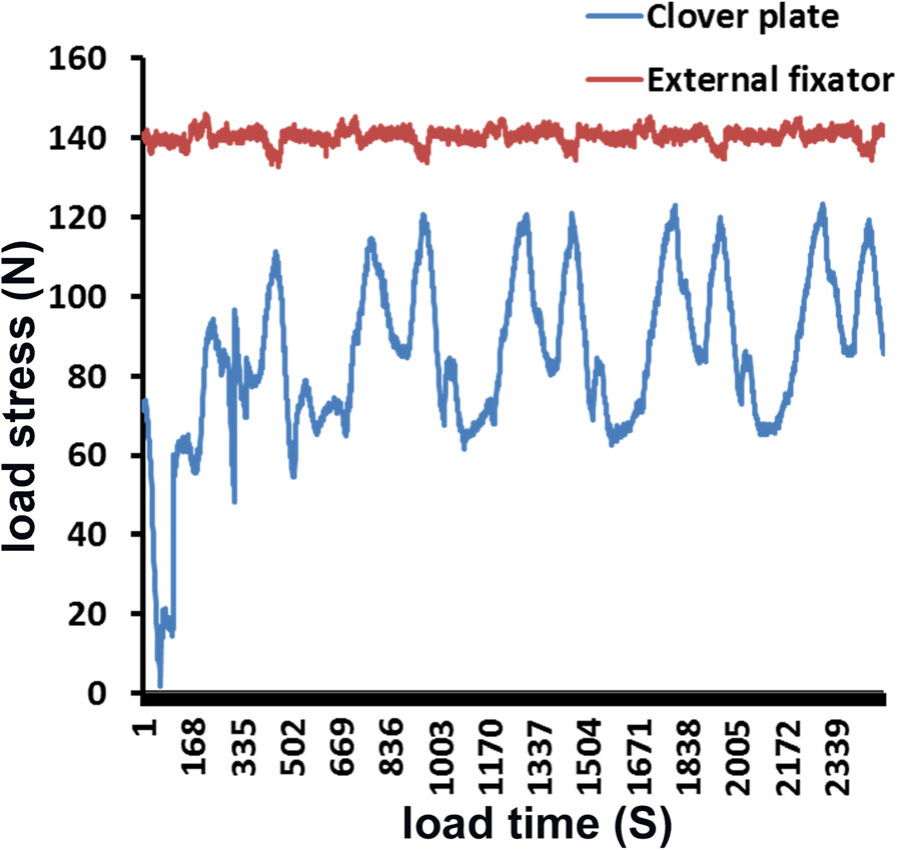
Comparison of resistance to load stress between the plate and the external fixator groups. It shows that the external fixator had a significantly more uniform load bearing comparing with control group (*P* < 0.05)

The bone density was 0.72 ± 0.3 g/cm^2^ in clover plate group and 0.73 ± 0.1 g/cm^2^ in the external fixation group. The ultimate bearing capacity of the clover plate was 120.21 ± 13.15 N, and the load bearing fluctuated with a forward spinning curve from 69.63 ± 25.16 N to 90.78 ± 17.18 N with humerus rotation, indicating that the clover plate was an eccentric fixation. Disalignment of the fixation from the spinning axis might lead to uneven loading and unstable fixation, with an ultimate displacement of 11.32 mm. The ultimate bearing capacity of the external fixator was 145.16 ± 17.42 N, which was significantly higher (*P* < 0.05) than the control group. And the load bearing fluctuated around 140 N at all points during humerus rotation, indicating a significantly more uniform load bearing than control group (*P* < 0.05; Fig. 6). The ultimate displacement was 10.48 mm, with no statistical significance comparing with the control group.

**Fig. 7:**
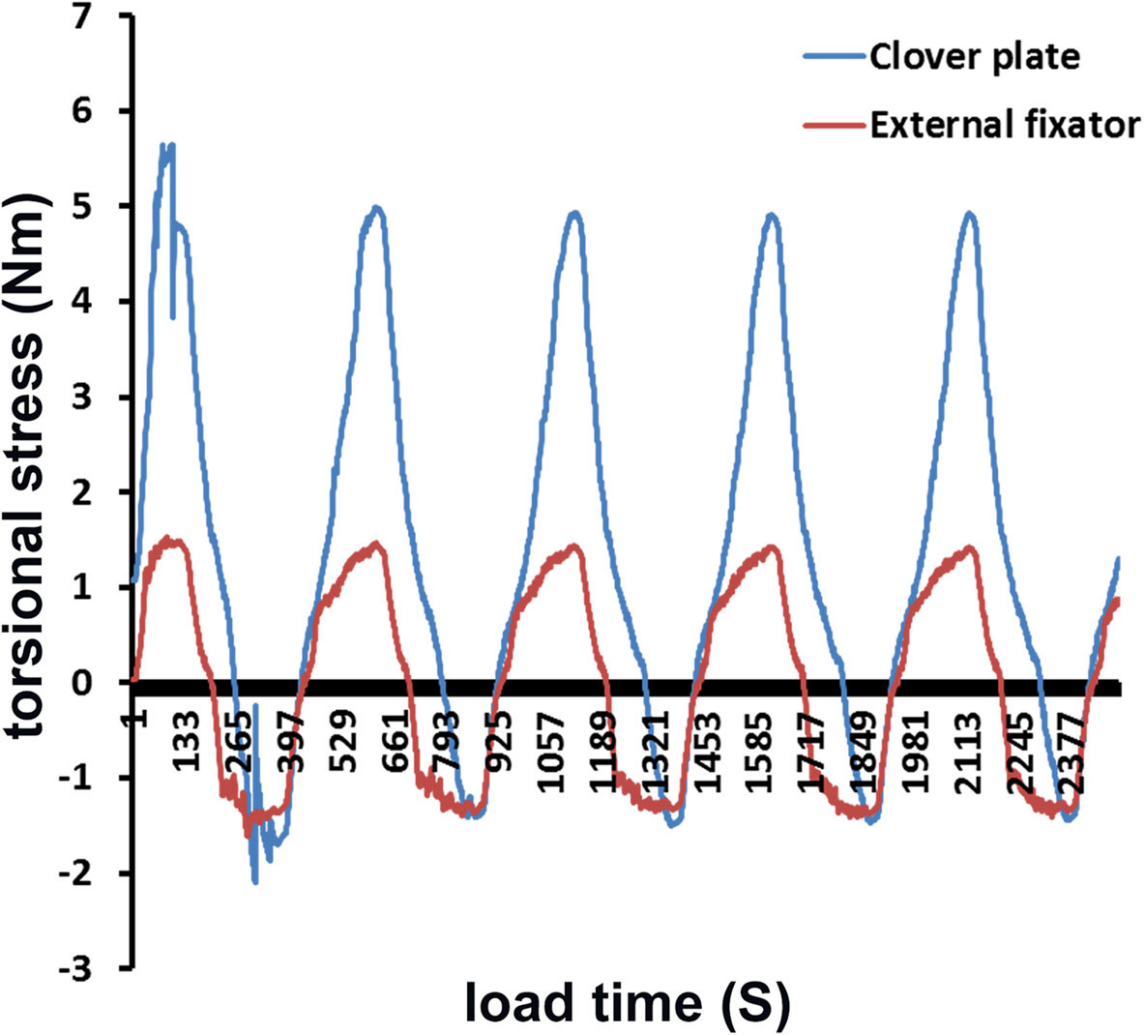
Comparison of resistance to torsional stress between the plate and the external fixator groups. It shows that the torque was significantly more uniform (*P* < 0.05) in the external fixator group

As for the resistance to torsional stress, the torque was significantly more uniform (*P* < 0.05) in the external fixator group (Fig. 7), with a slight fluctuation within 1 Nm. The clover plate group showed an eccentric fixation, with the torque fluctuating between 1 Nm and 5 Nm; torsional resistance was not uniform during rotation. The torque near the plate was greater (*P* < 0.05) than the test group, while the resistance to torsional stress beyond the plate was similar (*P* > 0.05) to the external fixator.

## Discussion

External fixation is still popular in China. Closed pinning is suitable for treating proximal humerus fractures in elderly patients, but may cause re-loss of fracture reduction in individuals with osteoporosis. Previously, autopsy studies have confirmed that closed pinning is likely to cause damage to neurovascular structures [17, 18]. With the newly developed fixator, fixation was achieved by intersecting pins through the broken ends, and the screw points were around the humeral shaft. The first pin was located at the insertion of the deltoid muscle, the second was in front of the humeral shaft and lateral edge of the caput longum musculi bicipitis brachii, and the third was at the apex of the greater tubercle of humerus. Thus, it may avoid the possibilities of neurovascular damage. To effectively fix the broken ends, Russo et al. introduced a new type of triangle fixation in the humeral head and metaphysis [19]. Our new fixator method was based on this triangle fixation’s principle: the three pins were inserted through the coronal, sagittal and frontal planes, which formed a solid triangle fixation. It showed satisfactory potentials in treating two-part humeral fracture in elderly patients with osteoporosis.

In the present study, the humerus was connected to the external fixator via 3 screws at the broken ends, ensuring good and stable fixation. This fixation design was based on Tension Guide Fixator (TGF)‘s principle [20]. The force of broken ends during shoulder motion could generate equal compression to the fractured bone through the pins, thus it may improve fixation stability and fracture healing. Besides, the external fixator can not only prevent the withdrawal of fixation pins, but also avoid humeral head varus which is most likely to occur in proximal humeral fracture [21].

Fixation mechanisms of internal plate are different from those of external fixator. In terms of parallel pinning fixation, traditional T parallel pinning has a longer arm than the plate, and its fixation strength is lower than that of plate. In the present study, intersection pinning was applied in the external fixator, with the pins fixed in bone cortex through the humeral shaft and head, which is similar to the intramedullary fixation system. In the resistance to load stress test, the external fixator showed greater load bearing and more steady values during humerus rotation than control group (*P* < 0.05). And torsional resistance was significantly uniform in the fixator group compared with the clover plate group (*P* < 0.01). Therefore, intersection pinning in the external fixator group is likely to yield more stable fixation. Besides, the external fixator can contribute to promote fracture healing due to reduced stress shielding effect of the plate [22].

The novel external fixator in this study has been used clinically in treating humerus surgical neck two-part fracture in China. As previously reported, this external fixator could allow minimal invasion, fast healing and early rehabilitation exercise. [23] But still, there are only few reports concerning the effect of the present novel external fixator, especially in the elderly patients with osteoporosis. It is unclear whether the novel fixator could provide satisfactory biomechanical stability for the target population. The results of the present study showed that it could offer higher load bearing and resistance to torsional stress when fixing two-part humeral fracture in elderly patients with osteoporosis. It has the potential of clinical application in the elderly with two-part humeral fracture and osteoporosis, which deserves further research.

There are some limitations in this study. Diagnosis of osteoporosis bases on measurements on living humans rather than on specimen. In this study we applied WHO criteria of bone osteoporosis in humerus specimen, which is a drawback. Secondly, single stress was applied in all biomechanical tests until fixation failure. Normally, the stress affecting the humeral head is composed of join forces of compression, torsional and shear force stresses generated by muscles and soft tissues around the shoulder joints. Besides, it may have different effects on each individual when combined with different neck shaft angles. Last, the stability of the external fixation construct was only compared to a non-locking plate; a comparison to a locking plate needs to be performed. The variation between the mechanical strength of the external fixator and a locking plate may be different. Nevertheless, the results in this study can still reflect the stability of the two fixation configurations in a certain degree.

## Conclusions

In the fixation of two-part humeral fracture in elderly patients with osteoporosis, intersection pinning of external fixator appeared superior to plate fixation in resistance to load and torsional stress. Thus, intersection pinning of external fixator might have clinical potential with the above advantages.
